# Clustering and Erratic Movement Patterns of Syringe-Injected versus Mosquito-Inoculated Malaria Sporozoites Underlie Decreased Infectivity

**DOI:** 10.1128/mSphere.00218-21

**Published:** 2021-04-07

**Authors:** C. M. de Korne, B. M. F. Winkel, M. N. van Oosterom, S. Chevalley-Maurel, H. M. Houwing, J. C. Sijtsma, S. Azargoshasb, E. Baalbergen, B. M. D. Franke-Fayard, F. W. B. van Leeuwen, M. Roestenberg

**Affiliations:** a Department of Parasitology, Leiden University Medical Center, Leiden, The Netherlands; b Interventional Molecular Imaging Laboratory, Department of Radiology, Leiden University Medical Center, Leiden, The Netherlands; c Department of Infectious Diseases, Leiden University Medical Center, Leiden, The Netherlands; University of Copenhagen

**Keywords:** *Plasmodium berghei*, fluorescent microscopy, malaria, motility, sporozoite

## Abstract

Malaria vaccine candidates based on live, attenuated sporozoites have led to high levels of protection. However, their efficacy critically depends on the sporozoites’ ability to reach and infect the host liver. Administration via mosquito inoculation is by far the most potent method for inducing immunity but highly impractical. Here, we observed that intradermal syringe-injected Plasmodium berghei sporozoites (^syr^SPZ) were 3-fold less efficient in migrating to and infecting mouse liver than mosquito-inoculated sporozoites (^msq^SPZ). This was related to a clustered dermal distribution (2-fold-decreased median distance between ^syr^SPZ and ^msq^SPZ) and, more importantly, a 1.4-fold (significantly)-slower and more erratic movement pattern. These erratic movement patterns were likely caused by alteration of dermal tissue morphology (>15-μm intercellular gaps) due to injection of fluid and may critically decrease sporozoite infectivity. These results suggest that novel microvolume-based administration technologies hold promise for replicating the success of mosquito-inoculated live, attenuated sporozoite vaccines.

**IMPORTANCE** Malaria still causes a major burden on global health and the economy. The efficacy of live, attenuated malaria sporozoites as vaccine candidates critically depends on their ability to migrate to and infect the host liver. This work sheds light on the effect of different administration routes on sporozoite migration. We show that the delivery of sporozoites via mosquito inoculation is more efficient than syringe injection; however, this route of administration is highly impractical for vaccine purposes. Using confocal microscopy and automated imaging software, we demonstrate that syringe-injected sporozoites do cluster, move more slowly, and display more erratic movement due to alterations in tissue morphology. These findings indicate that microneedle-based engineering solutions hold promise for replicating the success of mosquito-inoculated live, attenuated sporozoite vaccines.

## INTRODUCTION

Since 2014, the number of cases of malaria worldwide has remained at 200 million annually, leading to more than 400,000 deaths every year, with children in sub-Saharan Africa bearing the greatest burden (according to the World Health Organization’s World Malaria Report 2019 [https://www.who.int/publications/i/item/9789241565721]). Malaria’s high morbidity and mortality underline the pressing need for an effective vaccine to support control programs. Live, attenuated Plasmodium falciparum sporozoite (PfSPZ)-based vaccine strategies currently are in clinical development (PfSPZ vaccine, PfSPZ CVac, PfSPZ-GA1 [1]) and have the potential of inducing up to 100% sterile immunity (2, 3).

To make live, attenuated sporozoites amenable for large-scale immunization, the U.S.-based biotech firm Sanaria has developed tools to isolate, purify, and cryopreserve sporozoites for injection. Unfortunately, the dermal or subcutaneous injection of sporozoites provided suboptimal protective efficacy (4). Although much better efficacy was obtained when high numbers of sporozoites were injected intravenously (5), the intradermal, intramuscular, or subcutaneous administration routes of low numbers of sporozoites are preferred to facilitate global administration to infants in countries of endemicity at a low cost of goods. A better understanding of the differences between potent mosquito-inoculated sporozoites (^msq^SPZ) and unsuccessful needle- and syringe-injected sporozoites (^syr^SPZ) is needed to boost the development of practical and efficacious attenuated sporozoite vaccines.

The potency of attenuated sporozoite vaccines critically depends on the ability of the sporozoite to migrate to and infect the host liver. Transgenic luciferase-expressing sporozoites and bioluminescence-based visualization of parasites in mice provide a macroscopic imaging platform to study the liver-stage parasite burden after different routes of administration (6). These studies indicated that mosquito-inoculated sporozoites migrate to the liver much more efficiently than intradermally injected parasites (7–10). The subsequent development of fluorophore-expressing sporozoites and a sporozoite fluorescent-labeling approach has allowed for more detailed microscopic studies on the motility of individual sporozoites, both *in vitro* and in skin (11–13). Moreover, automated analysis of sporozoite motility now provides a platform to quantitatively study sporozoite motility under different conditions (14–17).

**FIG 1:**
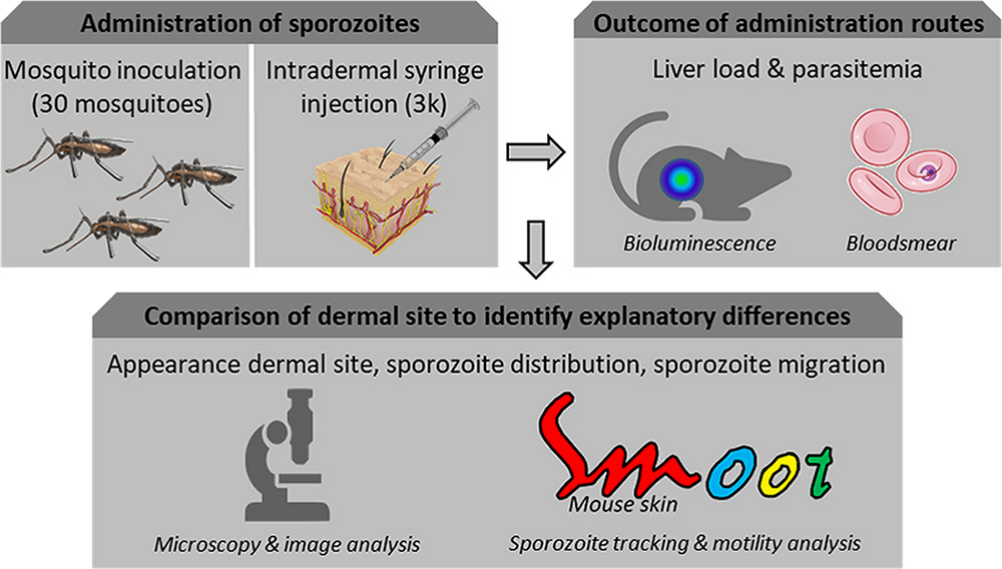
Study design. Sporozoites were administered via mosquito inoculation or intradermal syringe injection. Liver load was subsequently assessed by bioluminescence and blood smear patency. Detailed analysis of the appearance of the dermal site, the sporozoite distribution, and sporozoite migration behavior was performed to reveal underlying mechanisms of decreased infectivity.

We here aimed to unravel the factors underlying the difference in potency and infectivity between mosquito-inoculated and intradermal syringe-injected sporozoites (Fig. 1). For this, we microscopically examined the dermal site and quantitatively assessed the distribution of sporozoites and their motility patterns after inoculation of ^msq^SPZ and ^syr^SPZ through automated image analysis. We assessed liver-stage parasite burden through bioluminescence assays.

## RESULTS

### Infectivity of ^msq^SPZ and ^syr^SPZ.

**FIG 2:**
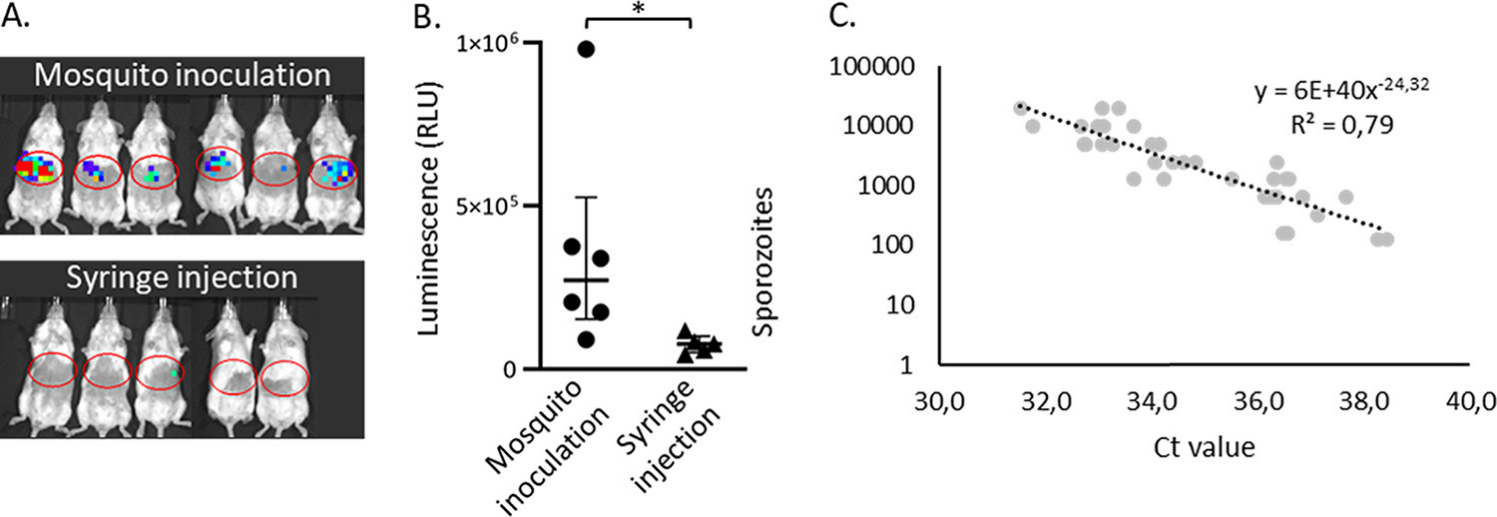
Outcomes for different administration routes. (A) *In vivo* images which show the liver load 44 h postinfection via 33 (IQR, 30 to 33) mosquito bites or intradermal syringe injection of 3,000 sporozoites. (B) Average of the luciferase activities in the liver 44 h after challenge by mosquito inoculation (median, 2.7 × 10^5^ RLU; IQR, 1.8 × 10^5^ to 3.7 × 10^5^ RLU) or intradermal syringe injection (median, 7.9 × 10^4^ RLU; IQR, 5.9 × 10^4^ to 8.5 × 10^4^ RLU) (*, *P* = 0.011; Mann-Whitney U test). (C) A calibration curve was generated based on a syringe-injected concentration range of sporozoites in skin (*n* = 3 in duplicate) to estimate the number of sporozoites delivered by 30 mosquito bites (median, 6,060 [2,203 to 13,481] sporozoites). Ct, threshold cycle.

At roughly equal numbers of administered ^msq^SPZ and ^syr^SPZ, the parasite liver loads of infected mice, assessed by bioluminescence imaging, were 3.5-fold higher in ^msq^SPZ mice (median, 2.7 × 10^5^ relative light units [RLU]; interquartile range [IQR], 1.8 × 10^5^ to 3.7 × 10^5^ RLU) than in ^syr^SPZ mice (median, 7.9 × 10^4^ RLU; IQR, 5.9 × 10^4^ to 8.5 × 10^4^ RLU; *P*, 0.011, Mann-Whitney U test) (Fig. 2). These results were in line with the results of previous reports (7). The prepatent period of blood-stage infection was on average 7 days after infection by mosquito bites. After syringe injection, 3/5 mice were still blood slide negative at day 9 after infection; the remaining two mice became positive at day 7 after injection.

Attempts to quantify the number of ^msq^SPZ by quantitative real-time reverse transcription-PCR (qRT-PCR) (Fig. 2) showed high variability in estimates (median, 6,060 ^msq^SPZ; range, 2,203 to 13,481 ^msq^SPZ), which was at least partly caused by technical variability inherent to DNA extraction from skin lysis samples. The estimated average number of ^msq^SPZ delivered did not significantly differ from the targeted 3,000 (*P*, 0.205, one-sample *t* test).

### Dermal site appearance.

**FIG 3:**
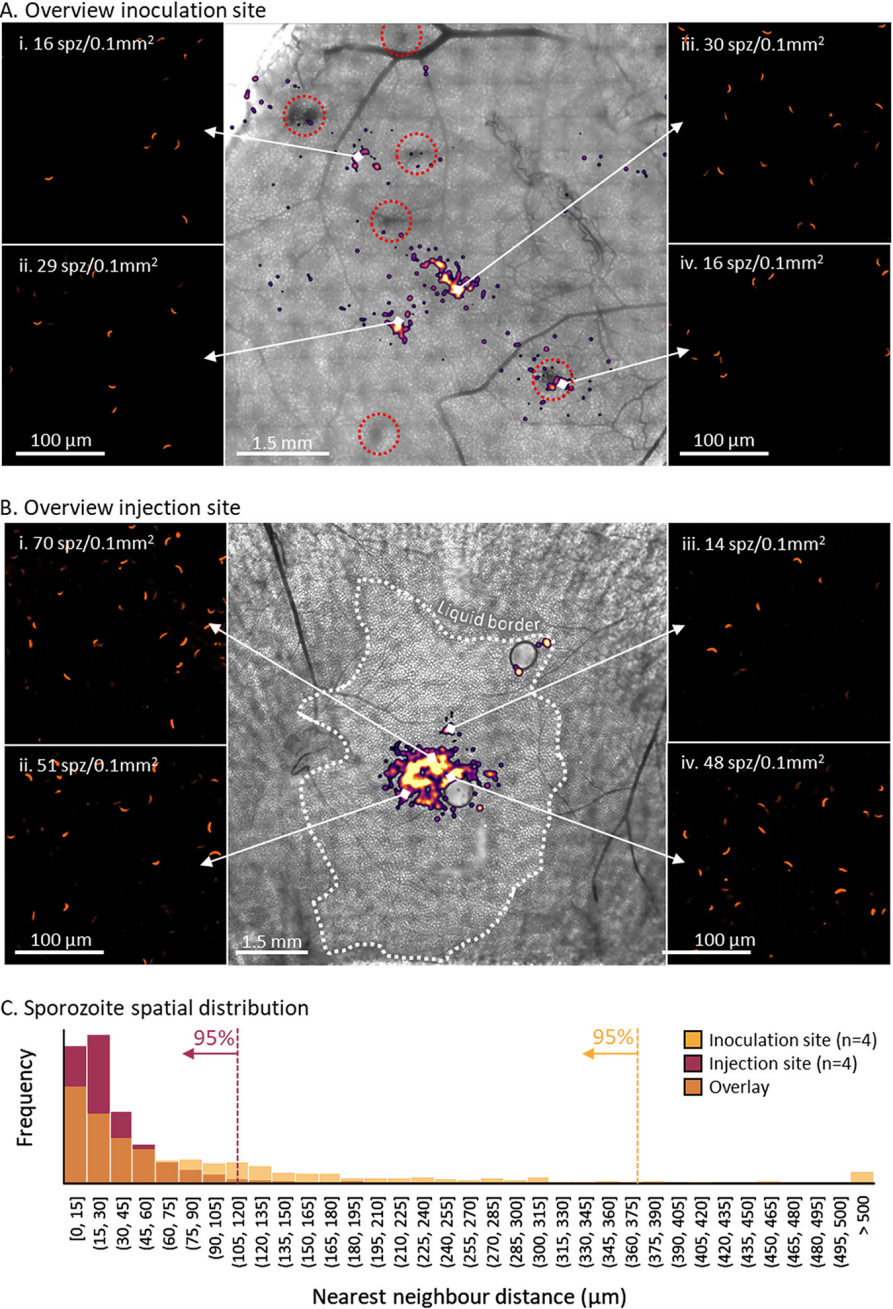
Overview of dermal site appearance. (A and B) Overview of the inoculation site after sporozoite delivery by mosquito (A) and of the injection site after sporozoite delivery by intradermal syringe injection (B), shown as an overlay of a bright-field images and the sporozoite distribution (pseudocolored, blurred fluorescent image), accompanied by zoom-in images showing individual sporozoites (i to iv). (C) Plot of the nearest-neighbor distance for ^msq^SPZ (yellow; median, 55 μm; IQR, 18 to 132 μm) or ^syr^SPZ (purple; median, 23 μm; IQR, 13 to 43 μm). The overlap of the two distributions is plotted in orange.

The dermal ^msq^SPZ and ^syr^SPZ sites were imaged over an average total depth of 124 μm (IQR, 103 to 131 μm) by confocal microscopy in order to visualize the sporozoite distribution. In general, ^msq^SPZ were distributed both individually and in clusters dispersed throughout the dermal tissue (Fig. 3A). We found that ^msq^SPZ dermal tissue contained multiple hematomas (median number per sample, 6.5). Interestingly we also found hematomas and ^msq^SPZ in the mouse peritoneum (see Fig. S1 in the supplemental material). Of the ^msq^SPZ identified, 9% were found within or in close proximity to the hematomas (example is shown in Fig. 3A, panel iv), which represented roughly 23% of the hematomas (6/26). Three quarters of ^msq^SPZ were found within a 255-μm radius of a blood vessel (Fig. S2).

10.1128/mSphere.00218-21.1 FIG S1 Peritoneum after mosquito bites. Overview of the peritoneum after sporozoite delivery by mosquito inoculation, shown as an overlay of a bright-field image and the sporozoite distribution accompanied by magnified images showing the individual sporozoites (i to iv). Download FIG S1, TIF file, 0.4 MB. Copyright © 2021 de Korne et al. 2021 de Korne et al. https://creativecommons.org/licenses/by/4.0/ This content is distributed under the terms of the Creative Commons Attribution 4.0 International license.

10.1128/mSphere.00218-21.2 FIG S2 Sporozoite-to-blood vessel distance. (A and C) Overview of the inoculation site after sporozoite delivery by mosquito (A) and the injection site after sporozoite delivery by intradermal syringe injection (C) shown as an overlay of a bright-field image, the sporozoite distribution (pseudocolored, blurred fluorescent image), and the segmented blood vessels (depicted in yellow). (B) Quantification of the sporozoite-to-blood vessel distance (*n* = 2 per condition). ***, *P* < 0.001; Mann-Whitney U test. Download FIG S2, TIF file, 0.2 MB. Copyright © 2021 de Korne et al. 2021 de Korne et al. https://creativecommons.org/licenses/by/4.0/ This content is distributed under the terms of the Creative Commons Attribution 4.0 International license.

In contrast, dermal sites containing ^syr^SPZ showed that the injected medium diffused throughout skin, with a single cluster of ^syr^SPZ in the center of this injection site (Fig. 3B). Zooming in on the ^syr^SPZ cluster showed that the ^syr^SPZ did not agglutinate (Fig. 3B, panels i to iv). We did not find hematomas in the ^syr^SPZ dermal tissue nor ^syr^SPZ in the peritoneum. On average, ^syr^SPZ were located further away from blood vessels, and three-quarters were found within a 504-μm radius of a blood vessel (Fig. S2) (*P* < 0.001, Mann-Whitney U test).

The sporozoite distribution was quantified according to their nearest-neighbor distance (NND), confirming the dispersed nature of ^msq^SPZ, with a median NND of 55 μm (IQR, 18 to 132 μm) and with 5% of the ^msq^SPZ further than 376 μm apart (Fig. 2C). In contrast, ^syr^SPZ were clustered at a median NND of 23 μm (IQR, 13 to 43 μm), with 5% of the ^syr^SPZ further than 112 μm apart.

**FIG 4:**
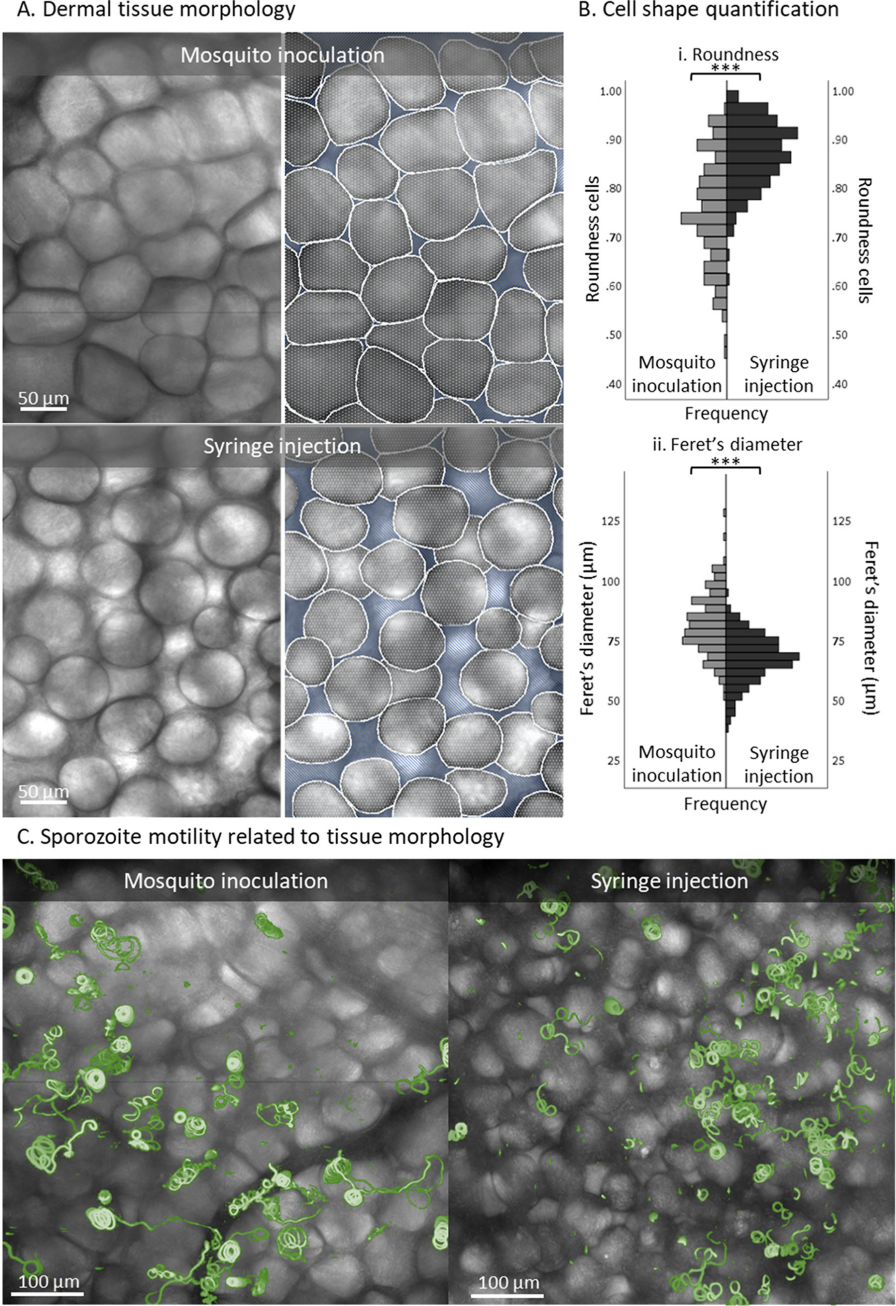
Magnifications of dermal tissue morphology after sporozoite delivery. (A) Magnification of the tissue morphology of the inoculation site after sporozoite delivery by mosquito and of the injection site after sporozoite delivery by intradermal syringe injection. Based on the bright-field images, the cells (depicted in white) and the interstitial space (depicted in blue) were segmented. (B) Quantification of the cell shapes found after mosquito inoculation (*n* = 164) and syringe injection (*n* = 203), using roundness (panel i) and Feret’s diameter (the longest distance between any two points along the cell membrane) (ii) as measures. ***, *P* < 0.001; independent sample *t* test. (C) Overview of the dermal site shown as an overlay of a bright-field image and a map of mosquito-inoculated and syringe-injected sporozoite tracks (depicted in green).

Zooming in on the morphology of the skin tissue, we found that after mosquito inoculation, cells remained densely packed, resulting in polygonal cells (mean roundness, 0.75 ± 0.11; Feret’s diameter, 82 ± 13 μm) (Fig. 4A and B). Conversely, after the syringe injection, the interstitial space between the cells was enlarged, leading to >15-μm gaps between cells and a change in cell shape toward significantly more rounded cells (mean roundness, 0.87 ± 0.07; *P* < 0.001, independent sample *t* test; Feret’s diameter, 68 ± 10 μm; *P* < 0.001, independent sample *t* test) (Fig. 4A and B). The projection of ^msq^SPZ and ^syr^SPZ tracks on top of bright-field images showing tissue morphology revealed that the altered tissue morphology was accompanied by altered movement patterns (Fig. 4C).

### Intradermal sporozoite motility. (i) Directionality.

**FIG 5:**
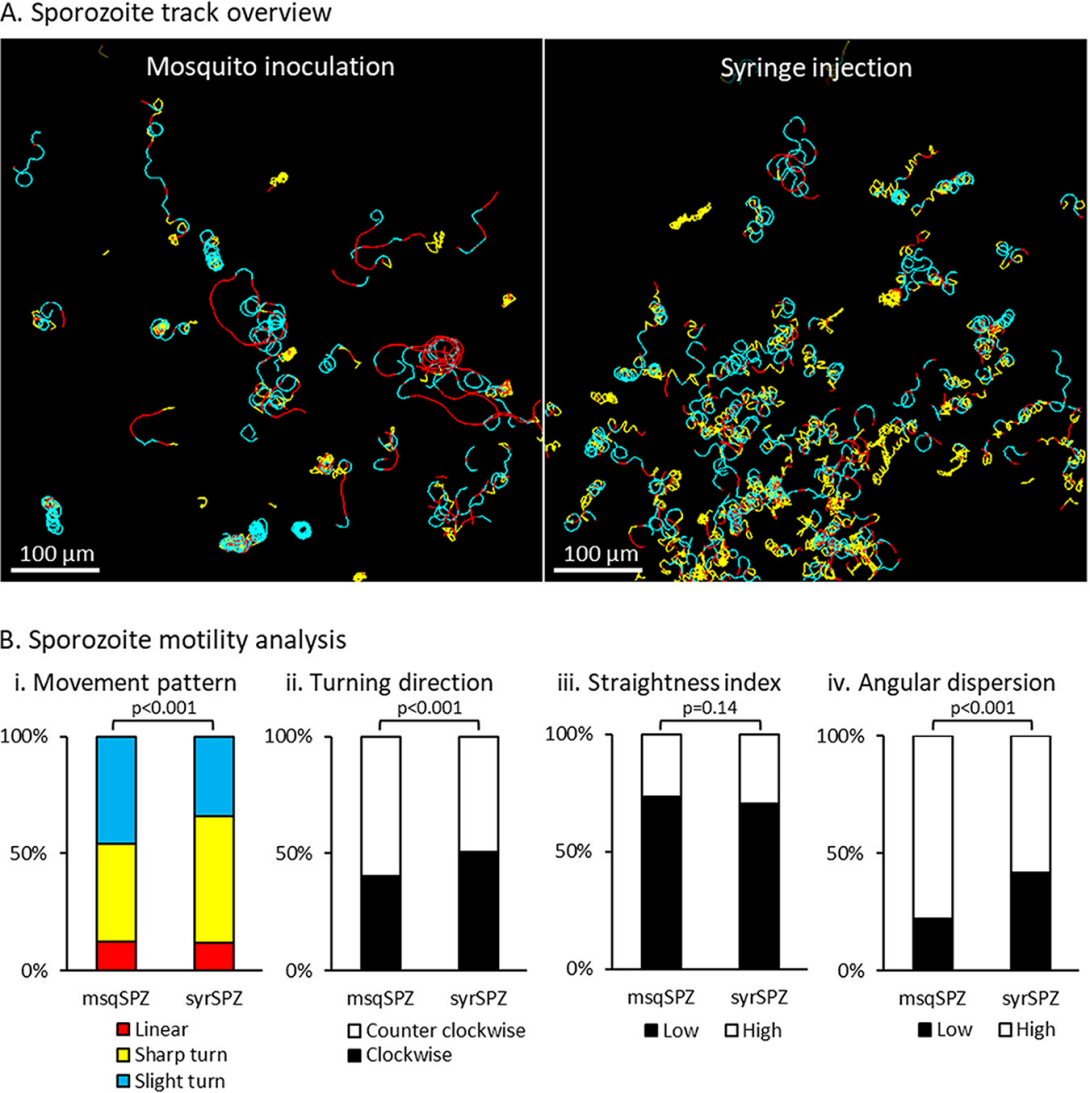
Movement pattern of sporozoites. (A) Overview of the inoculation site after sporozoite delivery by mosquito and the injection site after sporozoite delivery by intradermal syringe injection, shown as a map of sporozoite tracks, color-coded for movement pattern (sharp turns in yellow, slight turns in blue, linear movement in red). (B) Quantification of the different aspects of sporozoite motility after administration by mosquito or syringe: (i) the movement pattern distribution based at frames, (ii) the percentage of clockwise and counter clockwise segments, (iii) the straightness index of the tracks (low: <0.5, high: >0.5), and (iv) the angular dispersion of the tracks (low, <0.5; high, >0.5). *P* values obtained by chi-square test.

After delivery, the majority of sporozoites displayed tortuous movement through the dermal tissue; representative examples are shown in Fig. 5A. In total, the movement of 566 ^msq^SPZ and 1,079 ^syr^SPZ could be captured and analyzed. Both ^msq^SPZ and ^syr^SPZ were equally motile (respectively, 89% and 88%).

Following both administration routes, the tracks of the sporozoites were highly curved. Traditional motility measures, such as the mean squared displacement, were thus unsuitable to accurately describe the migration behavior of both groups of sporozoites (Fig. S3). Therefore, we included other parameters to investigate the tortuous migration behavior. Tracks were color coded for movement pattern, e.g., straight in red, slight turns in blue, and sharp turns in yellow. Both ^msq^SPZ and ^syr^SPZ showed equal numbers of turns and straight paths, with the percentage of turns at frame level at 88% for both samples (Fig. 5B, panel i). The percentage of sharp turns was somewhat decreased in ^msq^SPZ compared to that in ^syr^SPZ (slight/sharp turns by ^msq^SPZ, 46%/42%; ^syr^SPZ, 34%/54%; *P* < 0.001, chi-square test) (Fig. 5B, panel i). Analysis of the straightness index (Fig. 5B, panel iii) revealed a similarly high level of tortuosity in both conditions (median straightness index for ^msq^SPZ, 0.24; IQR, 0.09 to 0.53; for ^syr^SPZ, 0.28; IQR, 0.14 to 0.56; *P*, 0.14, chi-square test). Turns were made both clockwise (CW) and counterclockwise (CCW), with a slight preference for CCW in the ^msq^SPZ group (CW ^msq^SPZ, 40%; CW ^syr^SPZ, 51%; CCW ^msq^SPZ, 60%; CCW ^syr^SPZ, 49%; *P* < 0.001, chi-square test) (Fig. 5B, panel ii). Interestingly, the turn angle of ^msq^SPZ was much more consistent, described by angular dispersion (Fig. 5B, panel iv), than that of ^syr^SPZ (median angular dispersion of ^msq^SPZ, 0.74; IQR, 0.54 to 0.87; median angular dispersion of ^syr^SPZ, 0.58; IQR, 0.32 to 0.77; *P* < 0.001, chi-square test). In conclusion, both ^msq^SPZ and ^syr^SPZ traveled highly tortuous paths, although ^msq^SPZ exhibited less-sharp turns, a more consistent turn angle, and a predominance for the well-described preferred CCW turn angle.

10.1128/mSphere.00218-21.3 FIG S3 Mean squared displacement. (A and B) The mean squared displacement of the sporozoites after administration by mosquito inoculation or syringe injection is plotted. (A) All tracks are included. (B) The circular tracks are excluded (90% of mosquito inoculation frames, 95% of syringe injection frames). The mean squared displacement of the individual samples is plotted as a dotted line; a linear trendline is plotted as a solid line. Download FIG S3, TIF file, 0.04 MB. Copyright © 2021 de Korne et al. 2021 de Korne et al. https://creativecommons.org/licenses/by/4.0/ This content is distributed under the terms of the Creative Commons Attribution 4.0 International license.

### (ii) Velocity.

**FIG 6:**
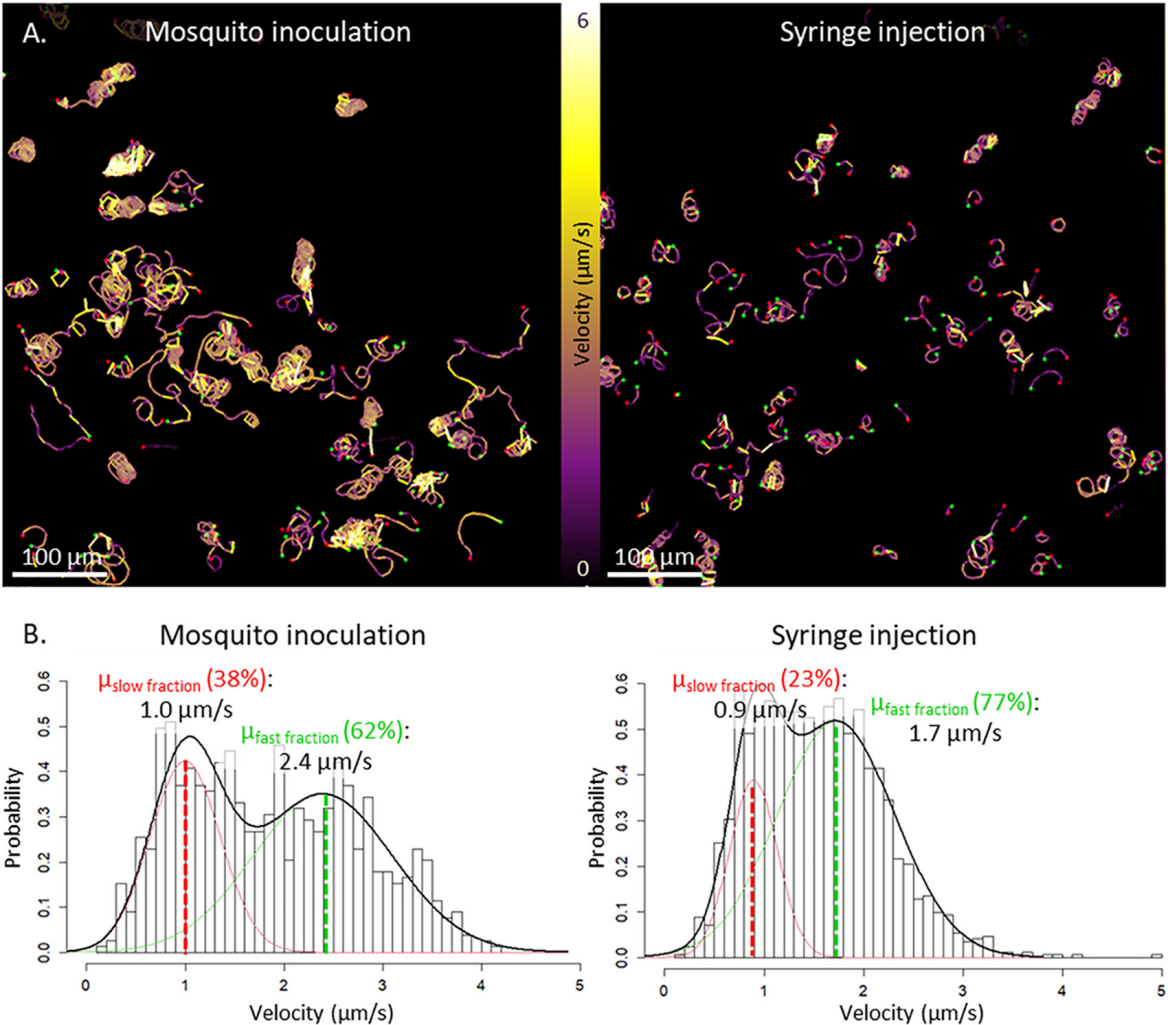
Velocity of sporozoites. (A) Overview of the inoculation site after sporozoite delivery by mosquito inoculation and the injection site after sporozoite delivery by intradermal syringe injection, shown as a map of sporozoite tracks, color coded for velocity (yellow sections correspond to high velocity, purple sections correspond to a lower velocity). (B) Distributions of the average track velocities, including a probability density function, with its mean determined using expectation-maximization-based fitting of a mixture of 2 normal distributions, one describing the slow-moving sporozoite fraction (depicted in red, accounting for 38% of the ^msq^SPZ and 23% of the ^syr^SPZ) and one describing the fast-moving sporozoite fraction (depicted in green, accounting for 62% of the ^msq^SPZ and 77% of the ^syr^SPZ).

Sporozoite velocity fluctuated along tracks (visualized by color coding in Fig. 6A), which was in line with earlier findings (14, 17–19). Plotting the average track velocities revealed a distribution that could be described with a mixture of two normal distributions (Fig. 6B). The first peaks were comparable for the two administration routes and contained the slow-moving ^msq^SPZ and ^syr^SPZ, with a mean velocity of, respectively, 1.0 ± 0.4 and 0.9 ± 0.2 μm/s. The second peak, containing the highly viable and rapid ^msq^SPZ and ^syr^SPZ, differed between the administration routes at a mean of 2.4 ± 0.7 μm/s for ^msq^SPZ and a mean of 1.7 ± 0.6 μm/s for ^syr^SPZ. Thus, the rapid ^msq^SPZ moved, on average, 1.4-fold faster than the ^syr^SPZ.

### Interplay between motility parameters.

**FIG 7:**
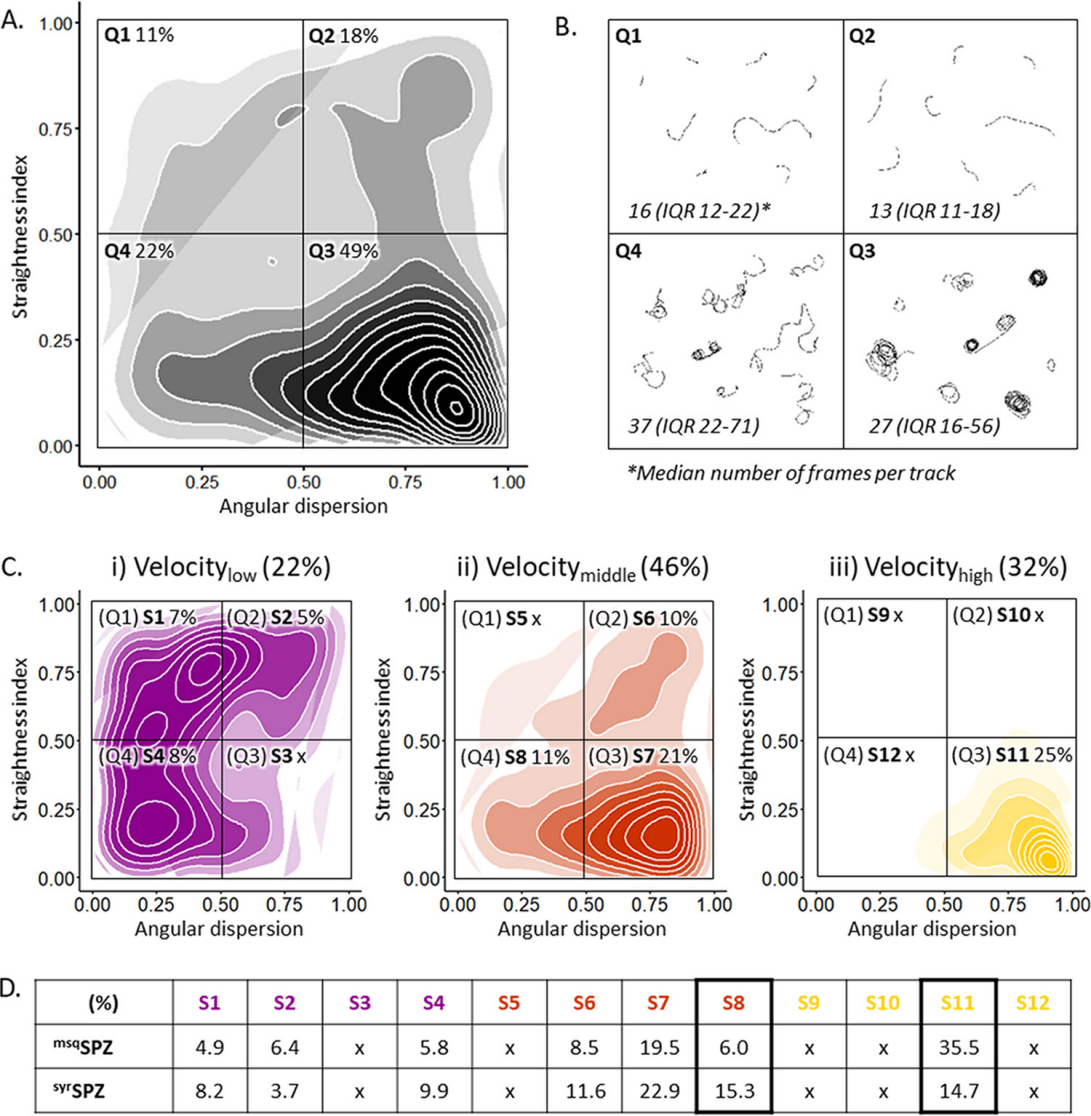
Interplay between motility parameters. (A) Density plot of all sporozoite tracks, including both ^msq^SPZ (*n* = 778) and ^syr^SPZ (*n* = 778), in a matrix of angular dispersion and the straightness index. (B) Representative example movement patterns associated with every quarter of the density plot. Group Q1, low angular dispersion, high straightness index (short, erratic tracks); Q2, high angular dispersion, high straightness index (short, straight tracks); Q3, high angular dispersion, low straightness index (consistently turning tracks); Q4, low angular dispersion, low straightness index (erratically turning tracks). (C) Density plot of all low (i)-, intermediate (ii)-, and high (iii)-velocity tracks on the same matrix of angular dispersion and the straightness index, resulting in 12 subsets (S1 to S12). Velocity categories are defined as slow (<1 μm/s, depicted in purple), intermediate (1 to 2 μm/s, depicted in orange), and fast (>2 μm/s, depicted in yellow). Percentages of the overall number of sporozoite tracks in every quarter of the density plots are given. (D) Comparison of the distributions (as a percentage of total tracks) for ^msq^SPZ and ^syr^SPZ tracks across subsets S1 to S12, with significant differences between S8 and S11 (*P* < 0.001, chi-square test).

To obtain a multidimensional view of sporozoite migration, we explored the relationship between tortuosity parameters. Based on high and low straightness index (SI) and angular dispersion (AD), the sporozoites’ tracks could be divided into four typical movement patterns: short erratic tracks (low AD, high SI), short straight tracks (high AD, high SI), consistently turning tracks (high AD, low SI), and erratically turning tracks (low AD, low SI) (Fig. 7A). Representative examples of tracks from these groups are shown in Fig. 7B. The majority of the tracks (49%) were classified as consistently turning (49%), which typically is the “default” movement pattern that sporozoites display *in vitro* (Fig. 7A).

We subsequently investigated the relationship between the four movement patterns and velocity. We found that consistently turning tracks were generally rapid sporozoites (median, 2.0 μm/s; IQR, 1.5 to 2.6 μm/s), whereas erratically turning sporozoites were considerably slower (median, 1.2 μm/s; IQR, 0.9 to 1.7 μm/s; *P* < 0.001, Mann-Whitney U test). Interestingly, these consistently turning rapid sporozoites were overrepresented within the ^msq^SPZ group compared to in the ^syr^SPZ group (2.4-fold difference), which was offset by a reciprocal increase in slower erratically turning sporozoites within the ^syr^SPZ group (2.5-fold difference) (*P* < 0.001, chi-square test) (Fig. 7D).

Lastly, we determined whether the distribution of the sporozoites as reflected by the NND influenced the tortuosity and velocity of their tracks. In general, the average NND of ^msq^SPZ was larger than that of ^syr^SPZ, as was observed by the analysis of the individual parameters (Fig. 3; Fig. S4). However, this difference was consistent among all different movement patterns (*P*, 0.52; interaction term ^msq/syr^SPZ × subsets, univariate general linear model), which suggested that the movement pattern or velocity of sporozoites was not dependent on their interindividual distance.

10.1128/mSphere.00218-21.4 FIG S4 Relation between motility parameters and nearest-neighbor distances (NNDs). The NNDs is plotted for the tracks split into 12 subsets (S1 to S12) based on four specific movement patterns (Q1 to Q4) (Fig. 7) and three velocity categories (1, <0.1 μm/s, depicted in purple; 2, 1 to 2 μm/s, depicted in orange; 3, >2 μm/s, depicted in yellow). The nearest-neighbor distance of the ^msq^SPZ is depicted in the darker color, and the NND of the ^syr^SPZ in depicted in the lighter color. Download FIG S4, TIF file, 0.04 MB. Copyright © 2021 de Korne et al. 2021 de Korne et al. https://creativecommons.org/licenses/by/4.0/ This content is distributed under the terms of the Creative Commons Attribution 4.0 International license.

Taken together, the ^syr^SPZ group contained more sporozoites that exhibited erratic movement at a slower speed, whereas the ^msq^SPZ group contained more sporozoites that circled consistently at high speed.

## DISCUSSION

Using confocal microscopy and dedicated sporozoite imaging software, we visualized sporozoites deposited by mosquito inoculation or syringe injection and assessed quantitative differences. We found that delivery by syringe injection decreases the infectivity of sporozoites by roughly 3-fold compared to mosquito inoculation, which is related to (i) a clustered distribution of ^syr^SPZ through the skin, with larger distances to blood vessels; (ii) a lack of hematomas, which are typically induced by mosquito bites; (iii) enlarged interstitial space due to syringe injection of fluid; and (iv) slower and more erratic migration patterns of ^syr^SPZ than those of ^msq^SPZ. Each of these parameters may impact the efficiency of sporozoite migration, blood vessel invasion, and ultimate liver infectivity and thus provide important insights into how to critically improve the delivery of sporozoite-based vaccines.

The dispersed distribution of ^msq^SPZ and the fact that a substantial proportion of ^msq^SPZ are deposited close to hematomas created by mosquito probing (20, 21) provide them with better odds of finding bloodstream access than with ^syr^SPZ. This is in line with the consistent circular motility of ^msq^SPZ, which has previously been associated with increased blood vessel engagement (13, 16). However, in previous publications, this engagement was generally related to deceleration, while in our study, consistent circular motility was related to high velocities (13, 16). Conversely, the slower erratic movement of ^syr^SPZ is most likely caused by the altered physical space as a consequence of fluid injection. Sporozoite movement is strongly guided by their three-dimensional environment; without any confinement, sporozoites *in vitro* display a continuous, preferential counterclockwise movement pattern (14, 18), while *in vivo*, skin structure redirects sporozoites to display much more complex patterns (15, 17). The role of physical confinement has been further supported by experiments whereby micropatterned *in vitro* environments were created that could induce specific movement patterns of sporozoites (22). We clearly found that the liquid which was coinjected with the ^syr^SPZ widened the interstitial space, which allows ^syr^SPZ to display erratic movement patterns.

Despite the fact that mouse skin does not fully replicate human skin with regard to skin thickness (mouse skin is <1 mm thick, and human skin is >2 mm thick [23]), as underlined by ^msq^SPZ deposition in the mouse peritoneum (length of mosquito proboscis,1.5 to 2.0 mm [24]), the remarkable differences between the ^msq^SPZ and ^syr^SPZ dermal sites provide important clues as to how to critically improve intradermal syringe injections of attenuated sporozoite vaccines. Particularly, a microneedle (patch) tattoo device or nanoliter injector may be useful not only to create the relevant sporozoite dispersion but also to decrease injection volume to the nanoliter range (25–29). In addition, laser-induced vascular damage can potentially mirror the hematomas induced by mosquito probing and enhance the blood vessel entrance of ^syr^SPZ (30). Recently, this concept was successfully applied to increase parasite loads in the liver after intradermal syringe injection (31).

Importantly, our study demonstrates that state-of-the-art imaging (analysis) techniques can provide valuable quantitative assessments of parameters affecting sporozoite migration. Our *ex vivo* setup combined with spinning-disk confocal microscopy and sporozoite tracking software enabled (i) the visualization of the sporozoite distribution throughout the inoculation and injection site (up to 100 mm^2^), whereas until now only one field of view (<0.5 mm^2^) was visualized during *in vivo* live imaging (13, 16, 32); (ii) the visualization of morphological tissue deformation as a result of fluid injection, previously acknowledged as an important parameter regarding transdermal drug delivery (33–35) but not yet visualized at a micron-level resolution; and (iii) a multidimensional analysis of sporozoite motility unveiling a remarkable interplay between motility parameters which were until now only studied independently (15–17). Further research is needed to study the role of other potential contributors not accounted for in this imaging study, such as their preprocessing of ^syr^SPZ (manual extraction from salivary glands in culture medium) and the effect of saliva inoculated by mosquitoes (14, 36, 37). Our quantitative assessment of parameters affecting sporozoite migration both indicates that engineering solutions that can better mimic mosquito inoculation should be explored and provides a readout needed to assess the potential of the suggested engineering solutions.

### Conclusions.

In conclusion, detailed microscopic imaging of the dermal site appearance and migration patterns of sporozoites revealed important quantitative differences between sporozoite administration via mosquito inoculation and intradermal syringe injection. These findings open new avenues for intradermal delivery of attenuated sporozoite vaccines with enhanced efficacy.

## MATERIALS AND METHODS

### Rodent experiments.

Mouse experiments were performed with female Swiss OF1 mice (6 to 7 weeks old; Charles River). All animal experiments were granted a license by the competent authority after advice on the ethical evaluation was received from the Animal Experiments Committee Leiden (protocol AVD1160020173304). All experiments were performed in accordance with the *Experiments on Animals Act* (38), the applicable legislation in The Netherlands, and in accordance with the European Union guidelines (39) regarding the protection of animals used for scientific purposes. All experiments were performed in a licensed establishment for the use of experimental animals (LUMC). Mice were housed in individually ventilated cages furnished with autoclaved aspen woodchips, a fun tunnel, a wood chew block, and nestlets at 21 ± 2°C under a 12-h/12-h light/dark cycle at a relative humidity of 55% ± 10%.

### Sporozoite production.

Naive mice were infected with the rodent malaria species Plasmodium berghei as described previously (7). The transgenic line 1868cl1 expressing mCherry and luciferase under the control of the constitutive HSP70 and eef1a promoters, respectively (*Pb*ANKA-mCherry_hsp70_ plus Luc_eef1α_; line RMgm-1320 [www.pberghei.eu]) was used. The infected mice were anesthetized, and Anopheles stephensi female mosquitoes were infected by allowing them to feed on gametocytemic mice, as described previously (40). The mosquitoes were kept at a temperature of 21°C and at 80% humidity until use.

### Sporozoite administration.

After the mice were anesthetized, P. berghei 1868cl1 sporozoites were administered using two different methods. (i) ^msq^SPZ were delivered by shaving the abdomen of the mice; 1 cm^2^ of skin was exposed for 15 min to around 30 infected mosquitoes (exact numbers are specified per experiment). Blood-fed mosquitoes were counted, and the presence of sporozoites in their salivary glands was confirmed using a quantitative analysis of luciferase activity after placing of the mosquitoes in a 20-μl drop of d-luciferin (8 mg/ml in phosphate-buffered saline [PBS]). (ii) ^syr^SPZ were obtained by manual dissection of the salivary glands of infected female Anopheles stephensi mosquitoes 20 to 24 days postinfection. The salivary glands were collected and homogenized to release sporozoites in Roswell Park Memorial Institute medium (RPMI medium; Thermo Fisher Scientific) enriched with 10% fetal bovine serum (Life Technologies Inc.). The free sporozoites were counted in a Bürker counting chamber using phase-contrast microscopy to prepare the injection samples. Directly after counting of the sporozoites (within 45 min of salivary glands being crushed), a 10-μl sample containing 3,000 sporozoites was administered by injection into the abdominal skin (within the same region as defined for exposure to mosquitoes) using an insulin syringe (Becton, Dickinson; Micro-Fine+, 0.5 ml, 0.30 by 8.0 mm, 30 gauge). The number of sporozoites delivered by exposure to 30 infected mosquitoes was considered consistent with intradermal delivery of 3,000 sporozoites via syringe injection based on data in the available literature (mean number of sporozoites inoculated per mosquito, 116 ± 28 [41]).

### Quantification of parasite liver load and prepatent period.

The liver stage of the P. berghei infection in 11 mice (6 mice challenged by 33 [IQR, 30 to 33] mosquito bites, of which 25 [IQR, 22 to 28] bites contained blood, and 5 mice challenged by syringe injection of sporozoites) was visualized, and sporozoites were quantified by measuring the luciferase activity in the liver at 44 h after the challenge with sporozoites using the IVIS Lumina II imaging system (Perkin Elmer Life Sciences). Before being imaged, the mice were shaved and anesthetized. IVIS measurements (exposure time, 120 s; binning factor, 16; aperture, f/1.2) were performed 8 min after subcutaneous injection of d-luciferin in the neck (100 mg/kg of body weight in PBS; Caliper Life Sciences). The liver load was quantified by measuring the total flux (photons per second) of a region of interest (ROI) covering the liver (the same ROI was used for all mice). Image analysis was performed using the Living Image 4.4 software (PerkinElmer Life Sciences). Infected mice were monitored for blood-stage infections with a Giemsa-stained blood smear until day 9 postinfection. The prepatent period (measured in days after sporozoite challenge) was defined as the first day at which blood-stage infection with a parasitemia of >0.5% was observed.

### Quantification of sporozoites by PCR.

Directly after sporozoite delivery by 32 (IQR, 32 to 33) mosquito bites, of which 18 (IQR, 17 to 19) contained blood, the skin of 4 exposed mice was cut out, snap-frozen, and stored at −20°C until further use. Parasite burden was measured by quantitative real-time reverse transcription-PCRs (qRT-PCRs). The DNA was extracted from the frozen skin using the QIAamp DNA microkit (Qiagen) by following the manufacturer’s instruction. Amplification reactions of each DNA sample were performed in PCR plates (hard-shell PCR plate, HSP9645; Bio-Rad) in a volume of 25 μl containing 12.5 μl PCR buffer (HotStarTaq master mix; Qiagen), 0.5 μl MgCl_2_ (25 mM), *Plasmodium*-specific forward and reverse primers (12.5 pmol; Plas-7F, 5′-GTTAAGGGAGTGAAGACGATCAGA-3′, and Plas-171R, 5′-AACCCAAAGACTTTGATTTCTCATAA-3′; Sigma-Aldrich), PhHV-specific (phocine herpesvirsus internal control) forward and reverse primers (15 pmol; PhHV-267S, 5′-GGGCGAATCACAGATTGAATC-3′, and PhHV-337AS, 5′-GCGGTTCCAAACGTACCAA-3′; Biolegio), a *Plasmodium*-specific FAM10-labeled detection probe (2.5 pmol; PP FAM [6-carboxyfluorescein], 5′-ACCGTCGTAATCTTAACC-3′; Biolegio), a PhHV-specific Cy5 double-labeled detection probe (1.25 pmol; PhHV-305TQ Cy5, 5′-TTTTTATGTGTCCGCCACCATCTGGATC-3′-BHQ2; Biolegio), and 5 μl of the DNA sample (dilution factor, 10×). Amplification consisted of 15 min at 95°C, followed by 50 cycles of 15 s at 95°C, 30 s at 60°C, and 30 s at 72°C. Amplification, detection, and analysis were performed with the CFX96TM real-time PCR detection system (Bio-Rad). A calibration curve to assess the sporozoite numbers in the mosquito inoculation samples was generated by analyzing skin samples injected with a dilution range of sporozoites (2-step dilution; start, 20,000 sporozoites; 10 samples, *n* = 3 performed in duplicate) (Fig. 2C).

### *Ex vivo* (fluorescent) imaging of the dermal site.

Immediately after sporozoite delivery, the exposed skin of 8 mice (4 administered ^msq^SPZ and 4 administered ^syr^SPZ) was excised, covered with a cover slip, and imaged using an Andor Dragonfly 500 spinning disk confocal lens on a Leica DMi8 microscope (Oxford Instruments) or a Leica true confocal scanning SP8 microscope (Leica Microsystems). The mCherry expressed by sporozoites was excited with the 561-nm laser. A 20× objective (HC PL APO 20×/0.75 IMM CORR CS2) was used, resulting in images of 617 by 617 μm. The experiments were performed within 1.5 h after tissue excision at room temperature.

To create overview images of the dermal site, up to 570 fields of view were stitched using the Andor imaging software Fusion (Oxford Instruments). Z-slices covering an average total depth of 124 μm (IQR, 103 to 131 μm) were imaged. Using the Fiji package for the open-source software ImageJ (42), the three-dimensional z-stack was reduced into a two-dimensional image using maximum-intensity projection (retrieves the level of maximum intensity along the *z* axis for each *x*,*y* position) and was converted into a binary image showing only the sporozoites. This image was further processed in two different ways: (i) the Gaussian blur filter was applied, and a pseudocolor image was created by applying the inferno-color lookup table in Fiji to visualize the location and density of the sporozoites; and (ii) the coordinates of the individual sporozoites in the ^msq^SPZ and ^syr^SPZ samples were determined, and the nearest-neighbor distance (the distance between the center points of neighboring sporozoites) was calculated using the ImageJ Nnd plugin. The blood vessels visible at the bright-field overview image of the dermal site were segmented using the Image Segmenter app available within MATLAB (MathWorks), and the distance from the sporozoite center to the nearest blood vessel was calculated. The number of sporozoites residing within or in close proximity to hematomas was determined using circular ROIs with a diameter of 650 μm and around the center of the hematoma. The shape of the cells visible at the zoom-in bright-field images (after mosquito inoculation, *n* = 164; after syringe injection, *n* = 203) was described by two shape descriptors available within ImageJ, namely, roundness (4 × [area]/π × [major axis]^2^), with a value of 1.0 indicating a perfect circle, and Feret’s diameter (the longest distance between any two points along the cell membrane).

### Sporozoite motility.

To analyze sporozoite motility, movies were recorded with frame rates of 35 to 40 frames/min and 200 frames per movie. Recording ^msq^SPZ samples (*n* = 6) yielded a total of 3,400 frames. Recording ^syr^SPZ samples (*n* = 8) yielded a total of 3,600 frames. Maximum-intensity projections of the recorded microscopy movies were generated using ImageJ. The motility of the sporozoites was analyzed using SMOOT_mouse skin_, an in-house-developed software program written in the MATLAB programming environment (MathWorks). This tool is an adapted version of the SMOOT_human skin_ (12, 17) and SMOOT*_In vitro_* (14) tools previously described. Via SMOOT_mouse skin_, the sporozoites could be segmented per movie frame, based on their fluorescence signal intensity, size, and crescent shape. The median numbers of pixel locations of the segmented sporozoites were connected into full sporozoite tracks.

First, the sporozoite tracks were characterized as motile or stationary based on their displacement, using a displacement cutoff of 21 pixels, which corresponds to the length of a sporozoite. Subsequently, the tracks of the motile sporozoites were subdivided into defined movement patterns: sharp turn, slight turn, and linear segments. Third, for the motile sporozoites, the mean squared displacement at frame level, the average velocity of their tracks, and the nearest-neighbor distance per track were calculated, and the tortuosity of the tracks was described via the straightness index (the ratio between the total length of the direct path between the start and the end of a track and the total length of the traveled path) and the angular dispersion (the deviation from the mean angle of the track). Finally, the interplay between the angular dispersion, straightness index, velocity, and nearest-neighbor distance was analyzed, for which the numbers of ^msq^SPZ and ^syr^SPZ tracks within the data set were equated using random sampling. Twelve subsets of sporozoite tracks were defined based on the angular dispersion (<0.5 and >0.5), straightness index (<0.5 and >0.5), and velocity (<1 μm/s, 1 to 2 μm/s, >2 μm/s).

### Statistical analysis.

The average and variability of the data were summarized using the mean and standard deviation (SD) for parametric data or the median and IQR for nonparametric data. For the comparison of groups, the difference between means or medians was assessed using, respectively, the independent sample *t* test and the Mann-Whitney U test. For the comparison of groups with a set value, the one-sample *t* test was used. For the comparison of the distribution of categorical data, the chi-square test was used, including a *post hoc* analysis based on residuals. A univariate general linear model was used to examine the relationship between a continuous variable and a categorical variable. *P* values of <0.05 were considered significant; in a case of multiple tests, the Bonferroni correction was applied to adjust the *P* value. All statistical tests were performed by SPSS Statistics (IBM Nederland BV). To compare the velocity distributions of both groups, the distribution was described by performing expectation-maximization-based fitting. The probability density function that could describe the sporozoite velocity distribution consisted of a mixture of 2 normal distributions. The package mixR (43) within the open-source R environment (44) was used to define the distribution parameters yielding the best fit.
