# Supplementary figures and images for: Clustering and Erratic Movement Patterns of Syringe-Injected versus Mosquito-Inoculated Malaria Sporozoites Underlie Decreased Infectivity
Source: mSphere. 2021 Apr 7;6(2):e00218-21. doi: 10.1128/mSphere.00218-21 (PMC8546700; doi:10.1128/mSphere.00218-21)

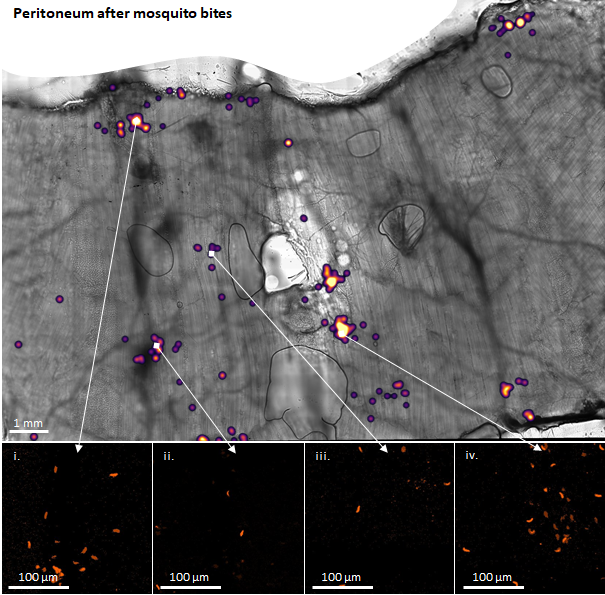

Supplement: FIG S1 [file msphere.00218-21-sf001.tif]

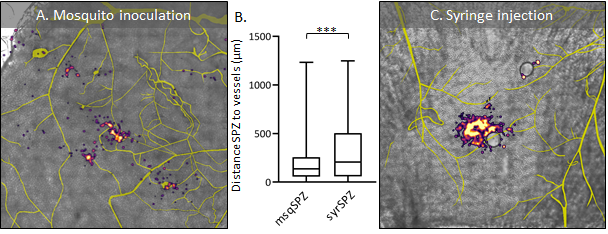

Supplement: FIG S2 [file msphere.00218-21-sf002.tif]

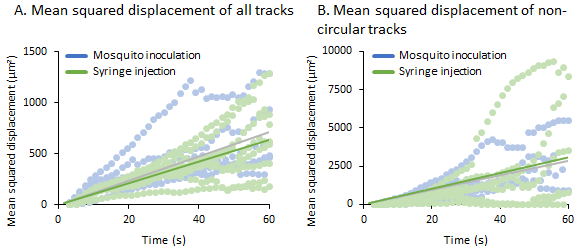

Supplement: FIG S3 [file msphere.00218-21-sf003.tif]

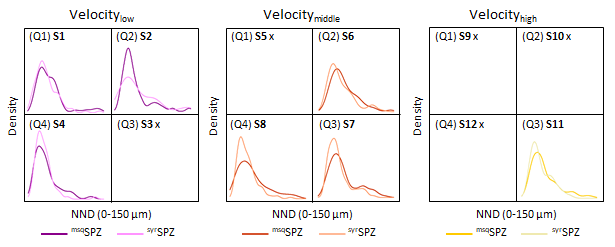

Supplement: FIG S4 [file msphere.00218-21-sf004.tif]
